# Hepatocellular Carcinoma: Tumorigenesis and Prediction Markers

**DOI:** 10.4021/gr2009.07.1304

**Published:** 2009-07-20

**Authors:** Cornelia Braicu, Claudia Burz, Ioana Berindan-Neagoe, Ovidiu Balacescu, Florin Graur, Victor Cristea, Alexandru Irimie

**Affiliations:** a Cancer Institute “I Chiricuta”, Cluj-Napoca, Romania; b University of Medicine and Pharmacy “Iuliu Hatieganu”, Cluj-Napoca, Romania

**Keywords:** Hepatocellular carcinoma (HCC), Tumorigenesis, Prediction markers

## Abstract

Hepatocellular carcinoma (HCC) is one of the most common malignancies. Although many advances have been made in the clinical study of HCC, the prognosis remains poor. Despite the discoveries in cancer biology in respect with physiological and pathological factors in relation to prognosis, HCC remains still a fatal disease due to late diagnosis. For improving the outcomes of patients with HCC, it is important to identify the factors predisposing to patient death. In recent years, based on cellular and molecular biology techniques, many tumor markers related to invasion, metastasis, recurrence and survival have been explored. However, routine biomarkers for the prediction of HCC evolution and prognosis are available in small number and less specific. These reviews focus on the recent advances in HCC tumorigenesis, revealing those biomarkers with prognosis significance or can be used for early detection.

## Introduction

Hepatocellular carcinoma (HCC) is one of the most common cancers with an incidence of 4 to 15 per 100,000 in Western countries, compared with 120 per 100,000 in Asia and Africa [1, 2]. Although advanced research in the clinical study of HCC has been made, the prognosis remain poor, HCC is one of the leading causes of worldwide cancer mortality, with an estimated number of 1 million annually deaths and a 5-year survival rate of less than 5% [3, 4]. Notably, men are about three to five times more likely to develop HCC than women [5]. Hormonal factors are underlining the hepatocarcinogenesis. Sex disparity characteristic in HCC could be attributed by both sex hormone pathways, with distinct role in each sex [6]. The increased activity of estrogens in female patients, which might protect them form carcinogenesis process [6, 7]. Therefore, up-regulation of the androgen pathways in male patients is considered to accelerate liver carcinogenesis [6]. Recently, it was demonstrated that estrogens can protect hepatocytes from malignant transformation via down-regulation of the secretion of IL-6 from Kupffer cells, a critical process in the diethylnitrosamine-induced HCC in mouse model [5, 7].

Chronic infections with the hepatitis B virus (HBV) and the hepatitis C virus (HCV) have been involved in about 80% of cases worldwide of HCC; also environmental risk factors, including alcohol abuse or dietary intake of food contaminant like aflatoxin B_1,_ were proven to be significant [8].

HCC is usually diagnosed at an advanced stage resulting in limited therapeutic options and poor prognosis. The identification of prognostic biomarkers is an important issue since such markers could facilitate early detection of HCC. Furthermore, such biomarkers could display potential therapeutic targets for HCC.

## Tumorigenesis

As for most types of cancer, hepatocarcinogenesis is a multi-step process involving different genetic alterations that ultimately lead to malignant transformation of the hepatocyte [1, 3]. A malignant cell phenotype is initiated when mutant hepatocytes produce mitogens that activate cellular receptors and intracellular signalling pathways. Genetic and molecular abnormalities generally associated with viral infection or due to the inflammatory condition represent an early step in hepatocarcinogenesis [9].

**Figure 1:**
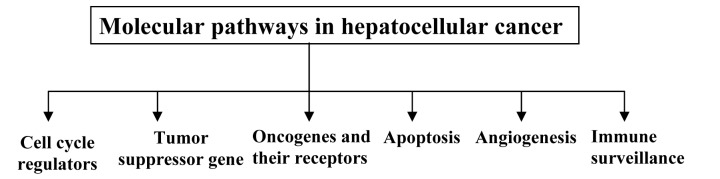
Molecular pathways involved in HCC tumorigenesis

HCC tumorigenesis includes alteration in cellular proliferation markers, cell cycle regulators, suppressor genes, oncogenes and their receptors, apoptosis related factors as well as modification of genes involved in angiogenesis and immune response as shown in Figure 1. However specific and molecular pathways that play pivotal role in the liver tumour development were not fully identified [4, 10-12].

## Epigenetics alteration pathways

Epigenetic alteration pathways may be a consequence of the normal aging process, persistent viral infection, and chronic inflammation. The epigenetic pathways are characterized by three main mechanisms: DNA hypermethylation leading to gene inactivation, DNA hypomethylation causing genomic instability, histone modifications affecting chromatin conformation [9, 13-15]. DNA methylation is the most frequent epigenetic alteration seen in mammalian genome, and it frequently mediates transcriptional repression [13]. DNA methylation occurs in different stages of liver disease (noncirrhosis, cirrhosis and HCC) [11]. It was observed that persistent viral infection, particularly HCV accelerates age-related methylation in the liver, suggesting the role in the pathogenesis of HCC [15]. Recent study demonstrated that both DNA hypomethilation and CpG hypermethilation are the dominant event during HCC development and progression [9, 11].

Therefore, epigenetic changes may serve as indicator or biomarker for screening of patients with an increased risk for HCC [14]. Therapeutic strategies being able to modify the methylation status or multikinase inhibitors of liver cancer cells and to target tumor suppressor genes may be highly beneficial in the treatment of human HCC [13, 16, 17].

## Tumor suppressor genes

Tumor suppressor genes represent genes that are likely to play a role negatively regulating cell growth. Loss or inactivations of these genes are associated with malignancy and carcinogenesis process. Apart from deletions and mutations, growing evidence has indicated that epigenetic alterations are implicated in inactivation of tumor suppressor genes [13, 18].

Like in other cancer types, DNA methylations of the tumour suppressor genes have been reported in HCC [13]. A correlation between hypermethylation expression and down-regulation of E-cadherin was reported in HCC. Decreased E-cadherin expression is correlated with epithelial-to-mesenchymal transition and metastasis and poor prognosis [13]. In addition, reduced or loss of E-adhering expression is mainly caused by aberrant CpG hypermethylation with a detectable frequency from 33% to 67%. Another study [19] described that loss of E-cadherin was closely associated with loss of heterozygosity of E-cadherin [14]. More in human HCC tissue was registered correlation among reactive species, E-cadherin regulation [13], Ras upstream inducer or downstream effectors [19].

Deletion at 17p and alterations of the p53 gene at 17p13 are common genetic changes reported in human cancers. The tumor suppressor gene p53 encodes a 53-kD nuclear phosphoprotein that acts as a transcription factor. The major functions of the gene are blockage of cell cycle progression in response to DNA damage, and mediation of DNA repair or apoptosis [18, 20, 21]. Mutations in the p53 gene abrogated its normal functions, leading to genomic instability and loss of growth control, p53 overexpression may be involved in determining the differentiation and the proliferative activity in many cancers including HCC [21-24]. Determination of p53 antigen and anti-p53 antibodies was proved to have a sensitivity of 41.1% in the diagnosis of HCC, and the over-expression of p53 in the serum or liver tissues of HCC patients prefigures the poorer prognosis and a shorter survival time [24].

## Cell cycle regulators

Disruption of the G1/S and G2/M check points leads to uncontrolled cell growth, resulting in the development and progression of cancers [25-27]. Overexpression of cyclin A, cyclin D, and cyclin E have been found correlate with the tumor relapse of human HCC, and are independent predictive markers for their recurrence and prognosis [27]. Liver cyclin proteins in general and cyclin D_1_ in particular may be considered as potential target for preventive and therapeutic strategies liked with the progression of the disease and culminating with HCC [25]. Amplification of the cyclin D_1_ gene and its overexpression was associated with aggressive forms of HCC [25, 26]. Experimental evidence also shows that cyclin D_1_ expression is sufficient to promote cell cycle progression in the absence of mitogen factor. In another in vitro study protein levels and activities of cyclin D1, E, Cdk4, cyclin A and Wee1 increased proportionally with the development of HCC, especially in the transition process from chronic hepatitis to HCC. Cdk6 and Cdk7 activities remained unchanged in the process from normal liver to HCC. These data suggest that the increase in Cdc2 kinase may play a role in the process from normal liver to chronic hepatitis, whereas the predominant increase in cyclin D1, Cdk4, cyclin E, cyclin A, and Wee1 suggests involvement not only in the process from normal liver to chronic hepatitis, but also during transition into HCC [27].

The p27 protein is a member of cyclin/cyclin-dependent kinase inhibitors involved in cell-cycle progression. p27 also promotes cell migration in metastatic HCC cells through the regulation of RhoA activity [28]. Reduced p27 expression correlates with poor prognosis meanwhile high p27 expression, correlated with prolonged survival, used as prognostic parameter for HCC [11, 13].

Recent findings demonstrate p73 accumulation in HCC, suggesting that p73 plays a role in the malignant phenotype. p73 expression status is related to prognosis of HCC patients [26, 27, 29].

## Oncogenes and their receptors

Proto-oncogenes encode a wide range of proteins products involved in the control of cell proliferation and differentiation, including growth factors, growth factors receptors, components of signal transduction pathways and transcription factors [30]. Aberrations of many oncogens were identified in HCC being associated with poor prognosis [12, 23].

The overexpression of epidermal growth factor (EGF) and epidermal growth factor receptors (EGFR) were observed in HCC [25, 31], being associated with late-stage disease, increased cell proliferation and degree of tumor differentiation [21]. EGFR can be considered as a marker for predicting the metastasis and recurrence of HCC [32, 33]. Because of high prevalence of EGFR overexpression in HCC, inhibitors of EGF-EGFR pathways are potential therapeutic agents [31, 33]. Studies of EGFR inhibitors in vitro, phase I or phase II studies were encouraging in HCC therapy [34, 35].

Transforming growth factor b1 (TGF-β1) is a potent growth factor inhibitor for most epithelial cells, involved in cell proliferation, migration, and differentiation and in response to injury [23]. TGF-β1 in human HCC cells displays significant intracellular expression, by autocrine stimulatory mechanism [2, 36]. It has been reported that TGF-β1 and TGF-β1 mRNA levels were significantly higher in the serum of patients with HCC compared to patients with non-malignant chronic liver diseases, the sensitivity was 89.5% and the specificity was 94% [36, 37]. It was also reported that TGF-β1 levels might increased in patients with cirrhosis, owing to decrease hepatic clearance. In addition, this biomarker is up-regulate in extrahepatic tumors, wound healing, angiogenesis and fibrosis, indicating the lack of disease specificity [37].

Hepatoma-derived growth factor (HDGF) is the original member of the HDGF family of proteins, which contain a well-conserved N-terminal amino acid sequence and nuclear localization signals (NLSs) in gene-specific regions other than the hath region and is essential for the induction of cell growth activity [36]. HDGF was more abundantly expressed in HCC than in the tumor free adjacent liver tissues in human and murine samples [36, 38]. An increased level HDGF in well-differentiated HCC compared with poorly or undifferentiated subtypes can have a potential prognostic utility for disease free and overall survival with HCC [36, 39, 40]. HDGF is an index of tumor multiplicity, being a sign of poor prognosis in HCC [36, 38, 39].

Hepatocyte growth factor (HGF) is present specifically in cancerous tissues, suggesting a correlation between this serine protease and hepatic malignancies [41]. Patients with inoperable HCC had higher levels of serum HGF than the healthy controls. Higher serum HGF levels for HCC patients are suggestive for a poor prognostic, indicating the active roles in disease progression [42].

Insulin-like-growth factor II (IGF-II) has been implicated in the pathogenesis of neoplasm of different tissues, including liver. This growth factor is believed to exert its effect during cellular proliferation [43]. A positive relationship of IGF-II levels and HCC progression was found in liver tissues and serum on experimental rats [44] and a limited number of studies on humans.

Deregulation of c-myc gene expression was frequently observed in experimentally induced HCC in rodents, as well as in primary human liver tumors [23]. Disease-free survival in patients with c-myc amplification is significantly shorter than in those without amplification [23]. Other nuclear oncogenes overexpressed are c-Ki-ras, c-Ha-ras, c-fos, c-fms and b-catenin with important role of HCC malignant phenotype [10, 30, 45, 46].

## Apoptosis

Apoptosis is a key mechanism causing cell death and organ diseases, failure of apoptosis is now understood to contribute to the development of human malignancies. Apoptosis rarely occurs in normal livers but increases in HCC, indicating that bcl-2 and bcl-xL expression play important role in regulating the apoptosis of normal liver and HCC. The relationship between bcl-2 related genes and HCC is still unclear [47, 48].

The expression of Fas and Fas ligand (Fas L) is associated with the prognosis of cancer patients. Fas expression level is significantly decreased in poorly differentiated HCC and of a large size tumor while Fas L expression in carcinoma cells is observed exclusively in moderately or poorly differentiated cases [26]. Sensitivity to TNF-related apoptosis-inducing ligand (TRAIL)-mediated apoptosis and the lysosomal pathway of cell death are features of cancer cells. Lysosomal permeabilization contributes to TRAIL-induced apoptosis of HCC cells and suggests that cFLIPL cytoprotection is, in part, due to p42/44 MAPK-dependent inhibition of lysosomal breakdown [48, 49].

Regulating action of apoptotic genes will deepen the understanding about the growth and development of HCC and offer valuable information to genetic therapy of HCC, thus enhancing the sensibility to radiotherapy and chemotherapy [47].

## Angiogenesis

Angiogenesis is a multistep process, physiological angiogenesis occurs during liver regeneration, leading to the formation of new blood vessel from pre-existing vasculature, meanwhile pathological angiogenesis occurs in HCC [4, 50]. Angiogenesis makes significant contribution to tumor growth, invasiveness, and metastatic potential of HCC. Differentially expressed angiogenesis genes and proteins were identified including,)platelet-derived growth factor receptor (PDGFR) vascular endothelial growth factor (VEGF), basic fibroblast growth factor (bFGF), matrix metalloproteinases (MMPs) and its inhibitors (TIMPs), angiopoitin-1 (Ang-1) angiopoitin-2 (Ang-1) have been evaluated and found to be related to HCC tumorigenesis and prognosis [50, 51].

PDGFR is overexpressed in endothelium of highly metastatic HCC [52]. The role of PDGFR in tumor angiogenesis is not clear; the correlation between its expression level and metastatic potential of HCC warrants further investigation [52, 53]. Over-expression of bFGF and their receptors is thought to be involved in carcinogenesis and, to clarify its clinical significance, the study of its blood level in cancer patients is important [44].

VEGF is considered one of the most important factors involved in tumor-associated angiogenesis, involved in neovascularization [51], development and/or progression of HCC, being associated with a poor survival [50, 51, 53]. HCC patients with level of serum and tissue VEGF overexpression have a lower survival rate [24]. In another study was demonstrated for the first time that retinoblastoma protein (pRb2/p130) is inversely correlated with VEGF expression and tumor aggressiveness. VEGF together with pRb2/p130 may act a diagnostic or prognostic indicator in HCC [24, 53, 54].

HCC tumour spread is partly dependent on neoangiogenesis [17, 54]. VEGF is considered one of the most important factors involved in neoangiogenesis, development and/or progression of HCC, being associated with a poor survival [50, 51, 53]. HCC patients with serum and tissue VEGF overexpression have a lower survival rate [24]_._ Oral multikinase inhibitor (e.g. Sorafenib, sunitinib) of the VEGF, PDGFR of downstream intracellular serine/threonine, is used with success in HCC therapy [17, 54].

Ang-1 and its antagonist, Ang-2, are ligands that regulate the Tie2 receptor. The Ang-2 gene is upregulated in the hypervascular type of HCC, being investigated in HCC [55]. Dominant Ang-2 expression against Ang-1 through Tie2 receptor in the presence of VEGF plays a critical role in initiating early neovascularization and transformation of noncancerous liver to HCC [53, 54, 55].

MMPs are proteins relate to the growth and infiltration of cancer cells. Expression of MMPs and TIMPs were investigated in vitro and surgically resected HCC tissues [56]. A high expression was observed for MMP-2, MMP-9, MT1-MMP and TIMP-2. Expression of MMP-7, MT2-MMP and TIMP-1 was found at a low frequency and a low amount in both cells and tissues. MMPs and TIMPs are involved in the progression of HCC [56].

## Immune surveillance

The inflammatory immune response of the host to viral antigens induces hepatocyte damage, which is followed by the regeneration of hepatocytes and the development of fibrosis and cirrhosis-important features in the pathogenesis of HCC [5, 7, 57]. Studies have demonstrated that cytokines can affect a multitude of cellular pathways. Although their normal function may be to keep the tumorigenic cascade at bay, disease states such as the presence of hepatitis viruses can alter cytokines to function as protumorigenic factors. These immune-related proteins are not only affecters of transformation and primary tumor phases, but they have also been shown to play a role in downstream pathways related to tumor progression, such as angiogenesis. Despite the known associations between cytokines and HCC, the full portrait of their mechanistic roles in this disease remains to be elucidated [7].

Mounting evidence indicates the involvement of cytokines in hepatocarcinogenesis [6, 57]. Interleukin-6 (IL-6) is a pleiotropic cytokine that plays a critical role in normal hepatic growth and liver regeneration following a reduction in hepatic mass. Concentrations of IL-6 in serum are increased in situations of chronic liver inflammation including alcoholic hepatitis, HBV and HCV infections, and steatohepatitis, conditions that may lead to development of HCC [7].

Serum levels of IL-6 and IL-10 are frequently elevated in patients with HCC but not in benign liver disease or non-HCC tumors [58, 59]. IL-6 and IL-10 may help identify a subset of HCC patients may serve as complementary tumor markers in these patients [7, 57, 60, 61]. These studies suggest that increases in IL-10 and perhaps other Th2 cytokines correlate with progression of HCC [57, 59, 60]. Proinflammatory IL-1β was elevated in HCC patients compared with healthy individuals [28, 57]. Serum IL-15 was higher in HCC indicating the degree of liver inflammation [62], TNF-α expression was elevated in HCC patients, especially those with recurrence. In addition, the levels of the TNF-α Rs (TNFαRI and TNFα RII) were higher in HCC patients [63]. In other studies, TNF-α level was lower in HCC tumor tissue versus the tissue surrounding the tumor and in HCC patients versus healthy individuals [57]. IFN-α was not detected in HCC, and was concluded that IFN-α may not play a large role in liver inflammation. The proinflammatory cytokines IL-12 and IL-2 are also increased in HCC. Th1 cytokines are mainly up-regulated in HCC [57].

Cox-2 is an isoform of cyclooxygenase, which is the key enzyme converting arachidonic acid to prostaglandins. Overexpression of Cox-2 affects many mechanisms involved in carcinogenesis, such as angiogenesis, inhibition of apoptosis, invasion and metastasis [64]. It has been shown that Cox-2 induces angiogenesis, which in turn aids tumor growth, invasion and metastasis. It was found positive correlations between Cox-2 and iNOS expression in HCV-positive HCC and this could be partially attributable to modulation of angiogenesis by Cox-2 [64, 65]. Overexpression of Cox-2 is generally higher in well-differentiated HCC compared with less-differentiated HCC or histological normal liver, with implication in the early stages of hepatocarcinogenesis [65], and increased expression of Cox-2 being significantly associated with shorter disease-free survival in patients with HCC. Cox-2 inhibitors might be effective in prevention of both cancer development and disease progression of HCC [64, 65]. Experimental studies on animal models with liver cancer have shown that both selective and non-selective Cox-2 inhibitors exert chemopreventive as well as therapeutic effects. However, the key mechanism by which Cox-2 inhibitors affect HCC cell growth isn’t yet fully understood [49, 50].

## Novel serum biomarker candidate for the enhanced detection of HCC

The overall increase in the incidence of HCC warrants efforts to prevent and treat more efficiently this disease. Therefore, early diagnosis, based on serum markers and the development of novel systemic therapies for advanced disease are very important [2, 4, 26].

Alpha-fetoprotein (AFP) is the only biomarker used for HCC diagnosis, however, its use in the early detection of HCC is limited, especially because about one-third parts of patients with HCC have normal levels of serum AFP [66]. Serum AFP is a marker that has low sensitivity and high specificity [8, 24, 67]. Other candidate markers are isoforms of AFP like lens culinaris agglutinin-reactive fraction of AFP (AFP-L3) and monosialylated AFP (msAFP) [68].

In a recent study serum transglutaminase-2 was presented as a novel histological/serologic candidate in HCC, especially for the individuals with normal serum AFP [64]. Determination of serum IGF-II in parallel with AFP as marker increases the diagnostic accuracy and sensitivity. IGF-II may be used as complementary tumor marker to discriminate HCC from cirrhosis [56]. Circulating interleukin-6 can be considered a promising tumor marker for HCC. In particular, the diagnostic value is significantly increased when is combined with other serum markers [69].

Glypican-3 (GPC3), a heparin-sulfate-proteoglycan was suggested to be serologic markers of HCC [70], results reached by three independent laboratories. Furthermore, due to the lack of correlation between serologic concentrations of GPC3 and AFP in HCC patients, the simultaneous use of both markers significantly increases the sensitivity of the test [71, 72].

Plasma levels of lipids, lipoproteins and apolipoproteins in HCC patients may reflect the status of hepatic cellular impairments, and decreased serum levels of cholesterol and apoAI may indicate a poor prognosis in HCC [73].

Des-γ-carboxyprothrombin (DCP) is an abnormal prothrombin that lacks γ- carboxylation of its glutamine residue. DCP is unable to bind calcium ion that is essential for its conformational transition and functional activity. DCP may differentiate HCC from non-malignant liver diseases, it can also identify HCC at an earlier stage [24].

Hepatoma specific gamma-glutamyl transferase (HS-GGT) was expressed in the development of HCC. The abnormal alteration of serum HS-GGT level is a sensitive tumor marker for HCC diagnosis, differentiation or prognostic marker, and the overexpression of GGT in HCC may be related to the hypomethylational status of GGT genes [42]. Some tumor markers, such as human cervical cancer oncogene and human telomerase reverse transcriptase mRNA, have also been indicated to have higher accuracies than AFP [24].

Human telomerase reverse transcriptase (hTERT) mRNA has been reported to be detectable in the serum of patients with different cancer type. The expression of hTERT mRNA in the serum of HCC patients is significantly higher than that in the serum of healthy adults with non-malignant hepatopathy. It has been reported that the sensitivity and specificity of hTERT mRNA in detecting HCC are 88.2% and 70.0%, respectively, indicating that the expression of serum hTERT mRNA is associated with the serum concentration of AFP, tumor size, and tumor differentiation degree, being a indicator of poor prognosis for HCC patients [24].

Squamous cell carcinoma antigen (SCCA), serine protease inhibitor, is physiologically expressed in the skin and other squamous epithelial cells. SCCA levels were significantly elevated in HCC patients, compared to patients with cirrhosis only or normal subjects [74]. SCCA is also over-expressed in serum from patients with HCC. SCCA was proved to have high sensitivity and low specificity [37].

Golgi protein 73 (GP73, also known as Golph2) is a resident Golgi-specific membrane protein expressed by biliary epithelial cells in normal liver. GP73 is up-regulated in HCC. Measurement of serum GP73 based on immunoblots revealed a sensitivity and specificity of 69% and 75%, respectively [8, 37].

Some other serum markers, such as: alpha-L-fucosidase, TGF-1, TNF-a, VEGF, IL-8, HGF are useful for diagnosis but none of these markers has been validated for clinical use [8, 24, 67].

The limitation of developing HCC markers is probably due to heterogeneity assay methods and result reporting, limited analysis of demographics and causes of liver disease as covariates in the expression of these biomarkers. In addition, these molecules need to be validated and cost-effectiveness especially those markers proposed as diagnostic or prognostic role [8]. Further studies are need to confirm the roles and to validate these biomarkers.

## Conclusions

In recent years many biological factors have shown association with the invasiveness of HCC in addition to their prognostic significance. These suggest new targeted biological therapy for HCC, antiangiogenic therapy using specific inhibitors for expressed angiogenesis genes or their receptors, methylation status of tumors suppressor genes and oncogenes. Other targets are serum proteins involved in inflammatory processes or apoptotic pathways.

The success in HCC treatment is influenced by early detection. Clearly, new biochemical markers are needed for HCC screening. AFP in simultaneous combination with other serum markers could enhance the sensitivity for HCC detection. The possibility of distinguishing cirrhosis as a high-risk group for HCC offers a hope for the early detection of HCC.

The prognostic factors or diagnostic biomarkers of plasma or serum are important trends that deserve attention. Developing new blood biomarkers will help develop more effective therapeutic strategy targeting key signalling. These novel markers should be easily measurable, reproducible and minimally invasive and need to be validated in large-scale studies in clinical practice**.**
